# Selective synthesis of *meta*-phenols from bio-benzoic acids via regulating the adsorption state

**DOI:** 10.1016/j.isci.2023.107460

**Published:** 2023-07-25

**Authors:** Xinze Du, Yumei Liu, Huixiang Li, Shenglin Liu, Xiaojun Shen

**Affiliations:** 1 State Key Laboratory of Catalysis, Dalian National Laboratory for Clean Energy, Dalian Institute of Chemical Physics, Chinese Academy of Sciences, Dalian 116023, China; 2 Beijing Key Laboratory of Lignocellulosic Chemistry, Beijing Forestry University, Beijing 100083, China; 3 University of Chinese Academy of Sciences, Beijing 100049, China; 4 Department of Chemistry, School of Chemical Engineering, Dalian University of Technology, Dalian 116023, China

**Keywords:** Chemistry, Organic chemistry, Green chemistry

## Abstract

Phenols are important building blocks widely applied in many fields. The pronounced orientational effect of the phenolic hydroxyl group makes achieving selective synthesis of *meta*-phenols challenging. Accessing *meta*-phenols needs lengthy synthetic sequences. Herein, we first developed a heterogeneous CO_2_-mediated CeO_2_-5CuO catalyst for decarboxylative oxidation of benzoic acids with a more than 80% selectivity to *meta*-phenols. This technology is based on a traceless directing group relay method. The CeO_2_-CuO catalysts with different Ce/Cu ratios exhibited controllable reaction selectivity between decarboxylation and decarboxylative oxidation. Spectroscopy experiments and computational studies showed the adsorption state of benzoic acid was found to be crucial for subsequent reaction pathways. The moderate adsorption on CO_2_-mediated CeO_2_-5CuO catalyst contributes to the distinct selectivity of phenol. Furthermore, the paddlewheel intermediate facilitates the synthesis of *meta*-phenols from benzoic acids. This traceless directing group method would promote the development of useful one-pot *meta*-substituted phenols from bio-based benzoic acids.

## Highlights

• One-pot synthesis of *meta*-substituted phenols over CeO_2_-CuO catalyst
• Decarboxylative oxidation reaction of biomass-based benzoic acids
• Improvement of phenol selectivity by regulating the substrate adsorption states

## Graphical abstract

**Figure:**
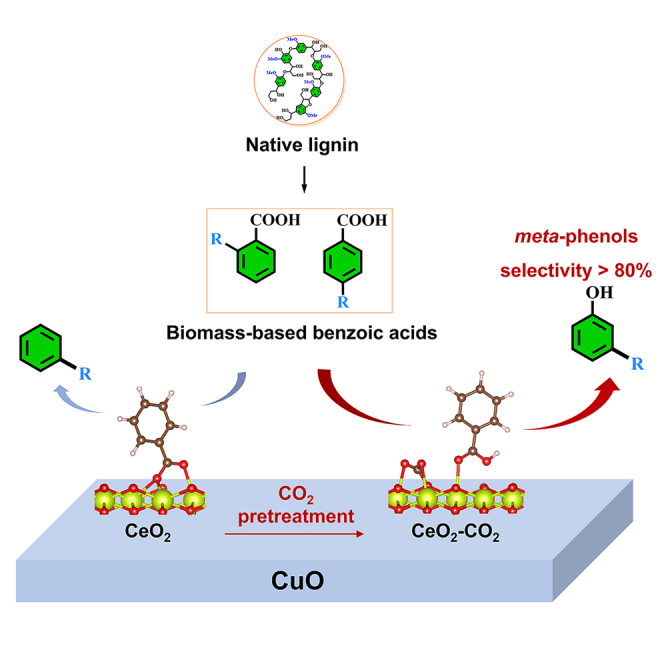

## Introduction

Phenolic chemicals are essential building blocks and functional moieties in a wide range of chemical and material industries, including pharmaceuticals, agrochemicals, and polymers, owing to their versatile nature and unique properties.^1^^,^^2^^,^^3^ Therefore, the development of strategies for the efficient selective functionalization of specific ring positions in phenols is of great interest to the chemical industry, offering promising avenues for the production of high-value products. Due to the excellent *ortho*/*para* orientation ability of the electron-donating phenolic hydroxyl group, various advanced technologies for the synthesis of the *ortho*/*para* functionalized phenol have been developed.^4^^,^^5^ This strong orientation effect, however, limits achieving selectivity in the synthesis of phenols substituted at the *meta* position. Over the past few decades, several effective strategies, including steric hindrance and directing-group control, have been developed and widely utilized for the efficient synthesis of *meta*-phenols.^6^^,^^7^^,^^8^^,^^9^ However, the practical implementation of these strategies is frequently limited by the need for interconversions involving the installation and removal of directing groups that can impede the formation of *meta*-substituted phenols. In addition, the competition between the orienting groups often leads to the formation of complex mixtures in molecules bearing multiple electronically or sterically active substituents. Moreover, the above strategies are generally homogeneous catalysis for the production of *meta*-phenols, which faces a great challenge in industrial manufacture, including separation from products, recycling, and preventing catalyst decomposition during the purification steps.^10^ Therefore, the establishment of a heterogeneous catalytic system that enables the direct synthesis of *meta*-phenols using readily available building blocks would be a highly attractive goal.

**Scheme 1:**
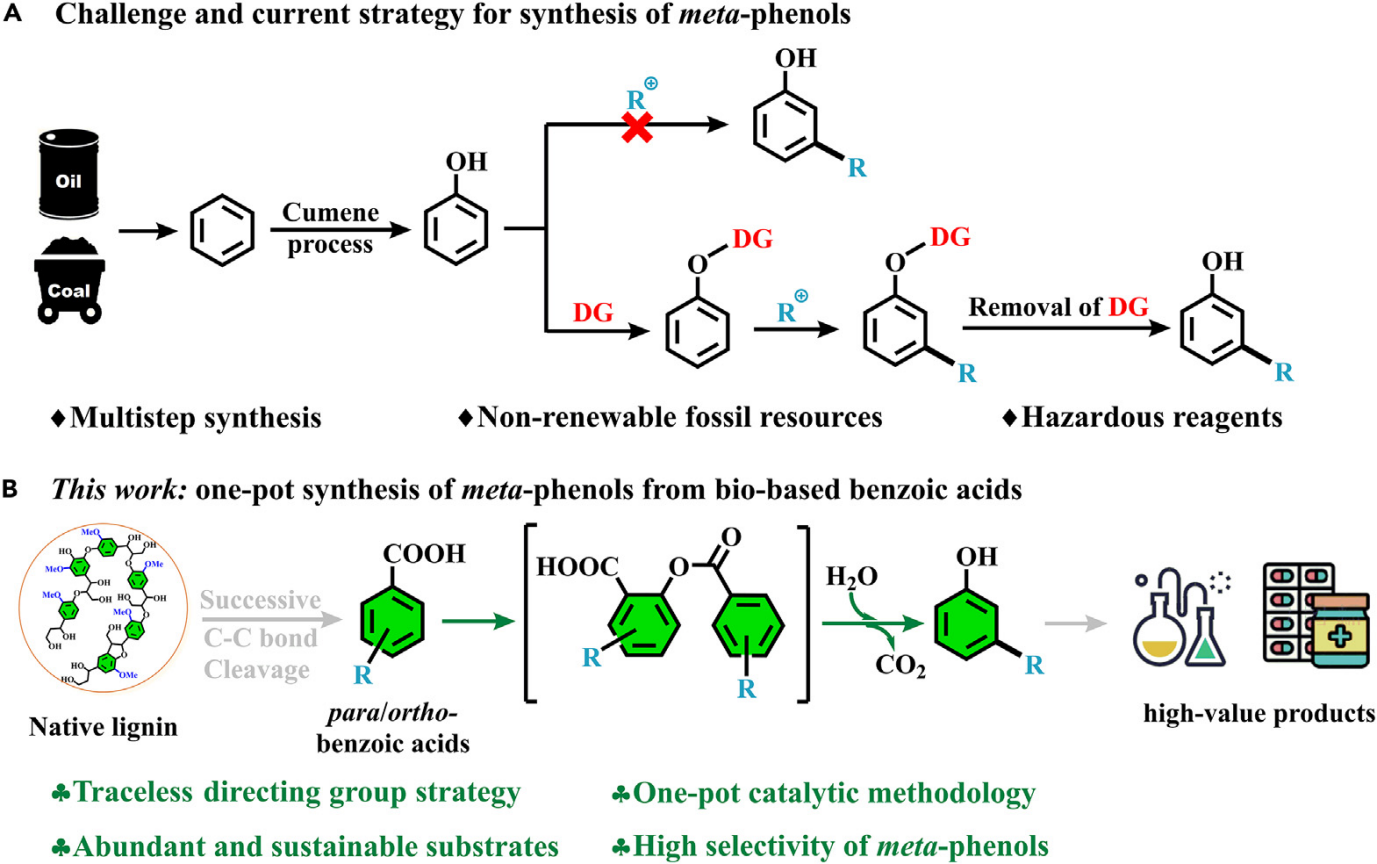
The challenge and strategies employed for the synthesis of meta-substituted phenols (A) Challenge and current strategy for synthesis of *meta*-phenols. (B) This work: one-pot synthesis of *meta*-phenols from bio-based benzoic acids.

On the other hand, benzoic acids (BAs) are abundant and stable chemicals. Recent studies show they can also be produced from the biomass platform^11^^,^^12^^,^^13^^,^^14^^,^^15^ or degradation of polystyrene.^16^ Therefore, an approach to transform BAs into phenols will be a potential method for commercial phenols production from biomass or plastics platforms. However, the implementation of this strategy is complicated by the high energy barrier associated with decarboxylation, as well as the challenges associated with achieving optimal reaction conditions for C–O bond formation.^17^ In 2021, Ritter et al. developed the first decarboxylative hydroxylation of BAs to synthesize phenols under mild conditions via radical decarboxylative carbometallation.^18^ However, this strategy is still used for the production of most *ortho*-phenols rather than *meta*-phenols. Recently, to overcome these challenges, researchers have developed a decarboxylative oxygenation method that selectively converted BAs to phenols through acid-promoted [1,2]-migration.^19^ To synthesize *meta*-phenols, *meta*-iodobenzoic acid was utilized to synthesize a range of meta-phenols via three distinct synthetic operations/purifications. Moreover, this process needs strong acid, oxidant, and environmentally unfriendly solvents, which is not favorable to the sustainable production of *meta*-substituted phenols. Following our recent development of copper-catalyzed oxidative cleavage of aromatic ketone and acid,^20^^,^^21^^,^^22^^,^^23^ we proposed that *meta*-substituted phenols could be formed from BAs by selective decarboxylative oxidation on Cu-based heterogeneous catalyst (Scheme 1).

For heterogeneous catalysts, the adsorption state of the substrate is crucial for the subsequent reaction pathway. Although CuO-based catalysts have been used for synthesizing phenol from BA (Dow phenol process),^24^^,^^25^^,^^26^^,^^27^ simple decarboxylation of BA to benzene and overoxidation of product, as side reactions, are still challenges for the high selectivity to phenol. The reported works usually focused on the screening of catalysts and optimization of operating factors.^28^^,^^29^^,^^30^ More importantly, BAs with substituents are more likely to decarboxylation to form arenes,^31^^,^^32^^,^^33^^,^^34^ which makes selective decarboxylative oxidation of substituted BAs remain technically elusive thus far. Therefore, we focus on how to suppress the decarboxylation as side reaction in this work. Considering that the formation of a unique paddlewheel intermediate in the decarboxylative oxidation pathway^35^^,^^36^^,^^37^ is quite different from the benzoate in the decarboxylation pathway,^31^^,^^32^^,^^33^^,^^34^ we suppose that it is a potential strategy to change the reaction pathway by regulating the substrate adsorption state. Herein, the adsorption state of BA on a CuO-based catalyst is regulated by the introduction of the CeO_2_ component to CuO and CO_2_ treatment. We find that the CO_2_-mediated CeO_2_-5CuO catalyst exhibits more than 80% selectivity to phenols in the decarboxylative oxidation reaction of BAs. Further spectroscopy experiments and computational studies illustrate the specific adsorption state of BA and verify its vital effect on product selectivity. Furthermore, the paddlewheel intermediate would generate *ortho*-salicylate benzoate, which facilitates the synthesis of *meta*-substituted phenols from *ortho* or *para*-substituted BAs.

## Results and discussion

### Catalytic performance of CuO-based catalysts

**Figure 1:**
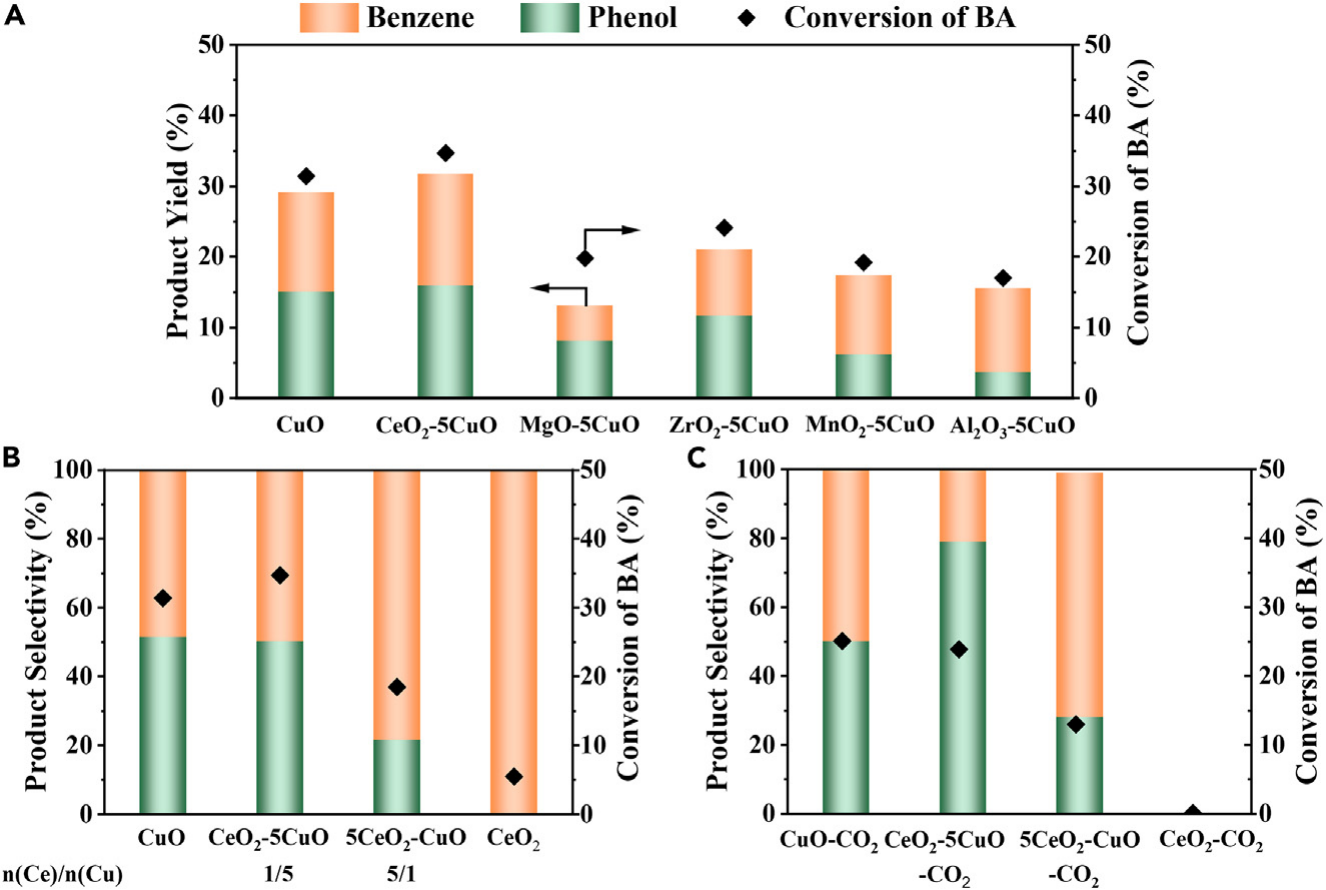
Catalytic performances of CuO-based catalysts in decarboxylative oxygenation reaction of BA (A) MO-5CuO. (B) CeO_2_-CuO with varying Ce/Cu ratios. (C) CeO_2_-CuO with varying Ce/Cu ratios after CO_2_ treatment. Reaction conditions: 1 mmol of Cu, 1 mmol BA, 20 mL H_2_O, 250°C, 12 h, Ar atmosphere.

To improve the phenol selectivity, we try to change the adsorption state of BA by introducing other metallic oxides (MO). In this work, CuO-based catalysts (MO-CuO) were initially synthesized by using an oxalate co-precipitation method (see STAR methods section for catalyst preparation and labeling). The catalytic performance of the decarboxylative oxygenation reaction of BA was evaluated at 250°C under an Ar atmosphere for 12 h. As shown in Figure 1A, CuO showed a 31.4% conversion of BA and 51.7% selectivity to phenol. The main by-product, benzene, was generated from the decarboxylation of BA. The conversion of BA and product yield were mainly limited by the additive amount of CuO-based catalyst. Then CuO-based mixed oxides (MO-5CuO, M = Mg, Ce, Zr, Al, Mn) were tested to identify the effect of surface acid-base properties on phenol selectivity. MgO-5CuO and ZrO_2_-5CuO exhibited higher selectivity (62.1% and 55.2%, respectively), but the conversion of BA decreased. Al_2_O_3_-5CuO and MnO_2_-5CuO cannot promote phenol selectivity. CeO_2_-5CuO provided a higher BA conversion (34.7%) and maintained the phenol selectivity (50.2%) as CuO. It seemed that introducing alkaline metal oxides to CuO may increase the phenol selectivity while decreasing the conversion of BA, but CeO_2_ exhibited the difference. Therefore, we further investigated CeO_2_-CuO catalysts with different Ce/Cu molar ratios (Figure 1B). With the increase of Ce/Cu molar ratios to 5 (5CeO_2_-CuO), the selectivity to phenol decreased, and benzene was the main product with 78.4% selectivity. Furthermore, CeO_2_ only showed a small benzene yield (2.6%). This may be attributed to the strong adsorption of the carboxyl group in BA at the basic sites of CeO_2_, leading to the decarboxylation reaction.^38^^,^^39^ Therefore, it can be concluded that the addition of CeO_2_ in CuO promotes the decarboxylation reaction of BA to benzene.

To cap the strong base sites, CeO_2_-CuO catalysts were treated with CO_2_ at 300°C for 1 h prior to the reaction (labeled as CeO_2_-CuO-CO_2_). As shown in Figure 1C, phenol selectivity increased on CeO_2_-5CuO-CO_2_ and 5CeO_2_-CuO-CO_2_ catalysts compared with those without CO_2_ treatment. Actually, these results were attributed to the suppression of the by-product benzene, as illustrated in Figures S1 and S2. In addition, only CO_2_ can be detected, and no other carbon-containing gas was found (Figure S3), which indicated that a decarboxylation reaction is involved in the process. Similarly, no product was detected catalyzed by CeO_2_-CO_2_. However, there was no obvious difference in catalytic activities between pristine CuO and CuO-CO_2_. This result suggested that the surface base properties of CuO cannot be regulated by CO_2_ treatment because there were no adsorption sites for CO_2_ on CuO. Overall, the CeO_2_-5CuO-CO_2_ catalyst showed the highest phenol selectivity (78.6%) and yield (15.8%) among the mentioned catalysts. The above results indicate that CO_2_ treatment may improve reaction selectivity by capping the base sites on CeO_2_.

### Characterization of CeO_2_-5CuO catalyst

**Figure 2:**
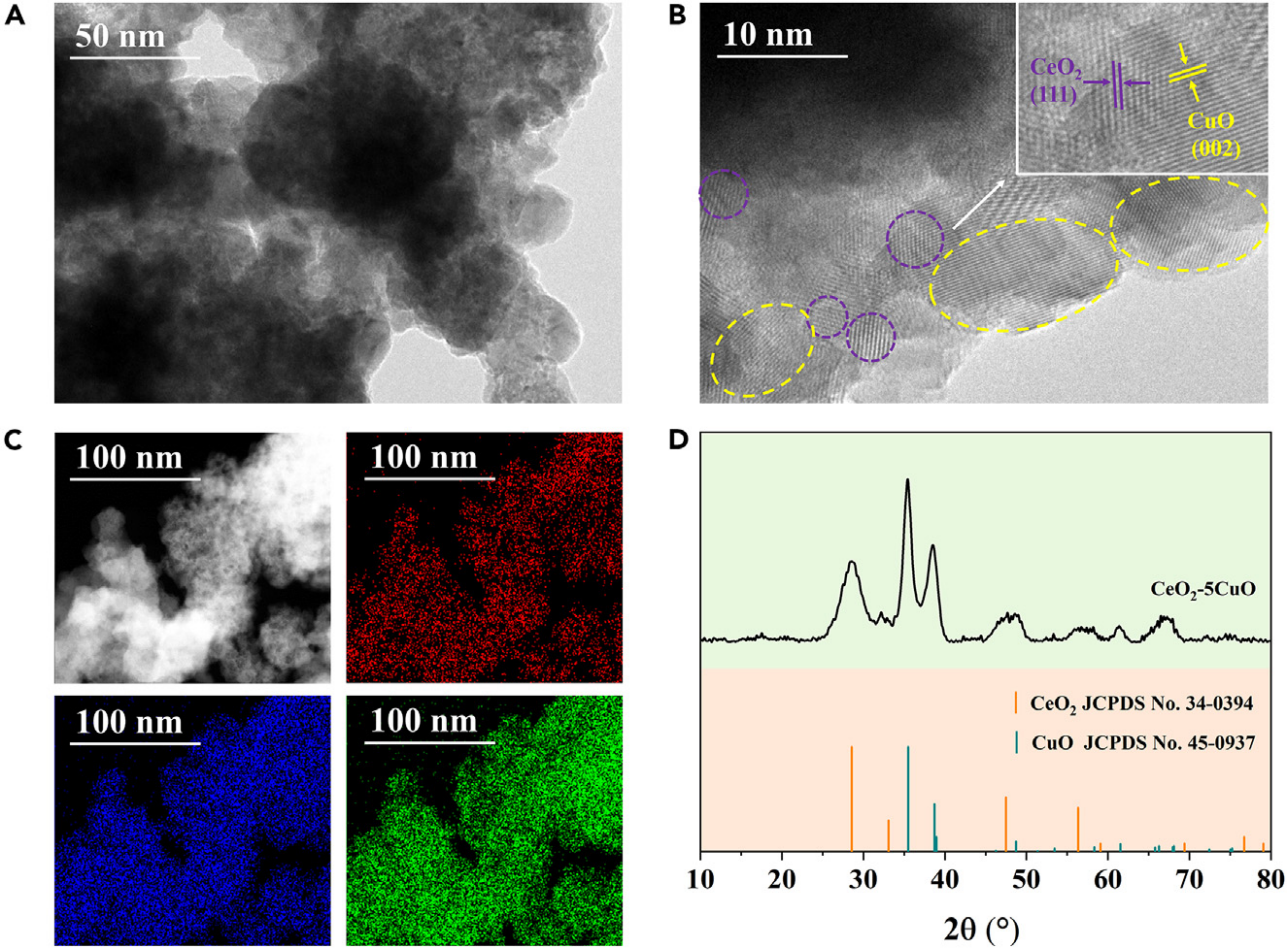
Characterization of CeO_2_-5CuO catalyst (A and B) TEM and HRTEM images. (C) EDS elemental mapping. (D) XRD pattern.

To study the components and structure of the CeO_2_-5CuO catalyst, we performed some characterizations. As shown in Figures 2A and 2B, the TEM and HRTEM images indicate that the CeO_2_-5CuO is mainly composed of irregular nanoparticles of CeO_2_ with a diameter of about 5 nm, which are dispersed on the CuO substrate. The preferentially exposed crystal planes are (111) of CeO_2_ and (002) of CuO, according to the corresponding lattice fringes. Elemental mapping images obtained from energy dispersive spectroscopy (EDS) in Figure 2C confirm the high dispersion of the component on the CuO substrate. Further X-ray diffraction (XRD) pattern (Figure 2D) confirms the CeO_2_ phase (JCPDS No. 34–0394) and CuO phase (JCPDS No. 45–0937) with the corresponding dominant crystal planes. X-ray photoelectron spectra (XPS) of the CeO_2_-5CuO catalyst show that the valence state of Cu is +2, and the valence state of Ce is +4, with a small part of +3 due to the existence of oxygen vacancies (Figure S4). The XPS results are consistent with those from TEM and XRD. To figure out the effect of CO_2_ treatment on CeO_2_-5CuO catalyst, we also studied the XPS spectra of CeO_2_-5CuO-CO_2_ (Figure S8). The Ce 3d XPS spectra showed that the percentage of Ce^3+^ was 25.2% in CeO_2_-5CuO (Figure S4), and decreased to 15.8% in CeO_2_-5CuO-CO_2._ This result was due to the adsorption of CO_2_ and then formation of CO_3_^2−^ at oxygen vacancies of CeO_2_, which has been widely reported before.^40^^,^^41^^,^^42^^,^^43^ Furthermore, the Lewis base properties of CeO_2_ were observed to be produced by the Ce^3+^ on the surface.^44^^,^^45^^,^^46^ Therefore, the surface base sites of CeO_2_-5CuO catalyst were finally modified by CO_2_ treatment as we expected.

### Spectroscopy and computational studies on substrate adsorption state

**Figure 3:**
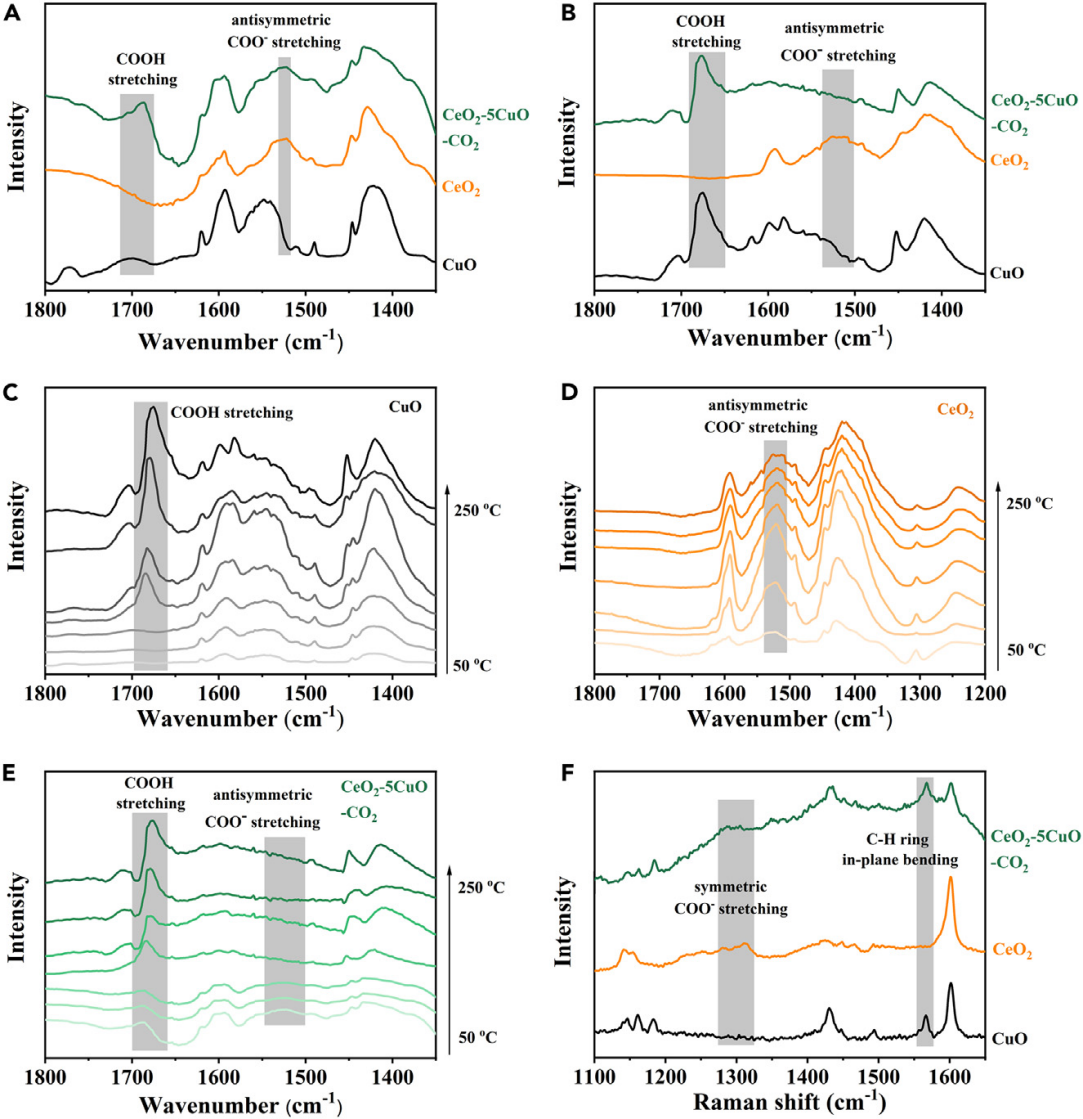
DRIFTS spectra of BA adsorbed catalysts (A) At 25°C. (B) At 250°C. (C) CuO at different temperatures. (D) CeO_2_ at different temperatures. (E) CeO_2_-CuO-CO_2_ at different temperatures. (F) Raman spectra at 25°C.

Diffuse reflectance Fourier transform infrared spectroscopy (DRIFTS) and Raman spectroscopy were then carried out to elucidate the specific BA adsorbates on the surface of different CeO_2_-CuO catalysts, which were crucial for the reaction mechanism. The CeO_2_-5CuO-CO_2_, instead of CeO_2_-5CuO, was chosen to study in contrast to CuO and CeO_2_, because CO_2_ treatment of catalyst is a determinant process for improving phenol selectivity. As illustrated in Figure 3, the IR peak located at 1678 cm^−1^ is identified as the asymmetric stretching mode of COOH of the absorbed BA, while the band observed at 1551 cm^−1^ corresponds to the antisymmetric stretching vibration of COO^−^ of the absorbed benzoate.^47^^,^^48^^,^^49^^,^^50^^,^^51^^,^^52^ The DRIFTS spectra at 25°C (Figure 3A) showed that both signals of COOH and COO^−^ were observed on CeO_2_-5CuO-CO_2_, but only COOH signals were observed on CuO, and only COO^−^ signals on CeO_2_. The results indicate that the forms of absorbed BA on CuO and CeO_2_ are totally different. The formation of benzoate on CeO_2_ is reported to result from the strong adsorption of BA on base sites over CeO_2_.^46^^,^^53^^,^^54^ The spectra of CeO_2_-5CuO-CO_2_ at 25°C seem to be a combination of CuO and CeO_2_, but results at 250°C (Figure 3B) show much different. The signals of COO^−^ were not detected on CeO_2_-5CuO-CO_2_ at 250°C. To further confirm the results, DRIFTS spectra at different temperatures of CuO (Figure 3C), CeO_2_ (Figure 3D), and CeO_2_-5CuO-CO_2_ (Figure 3E) were studied. The signals of COO^−^ on CeO_2_-5CuO-CO_2_ gradually weaken with the increase in temperature, and completely disappear at 200°C. But the signals of COO^−^ on CeO_2_ showed no obvious change. The signals of COOH were both observed on CuO and CeO_2_-5CuO-CO_2_. In addition, the Raman spectra verified the existence of COO^−^ on CeO_2_ and CeO_2_-5CuO-CO_2_ at 25°C (Figure 3F). Summarizing the information of the DRIFTS and Raman spectra, it could be deduced that the benzoate is easy to form on CeO_2_ and hold at 250°C due to the strong adsorption, while the adsorbed BA maintains the molecular state on CuO. The CeO_2_-5CuO-CO_2_ catalyst shows moderate adsorption of BA, which may be attributed to the combined effect of introducing CeO_2_ component and CO_2_ treatment.

**Figure 4:**
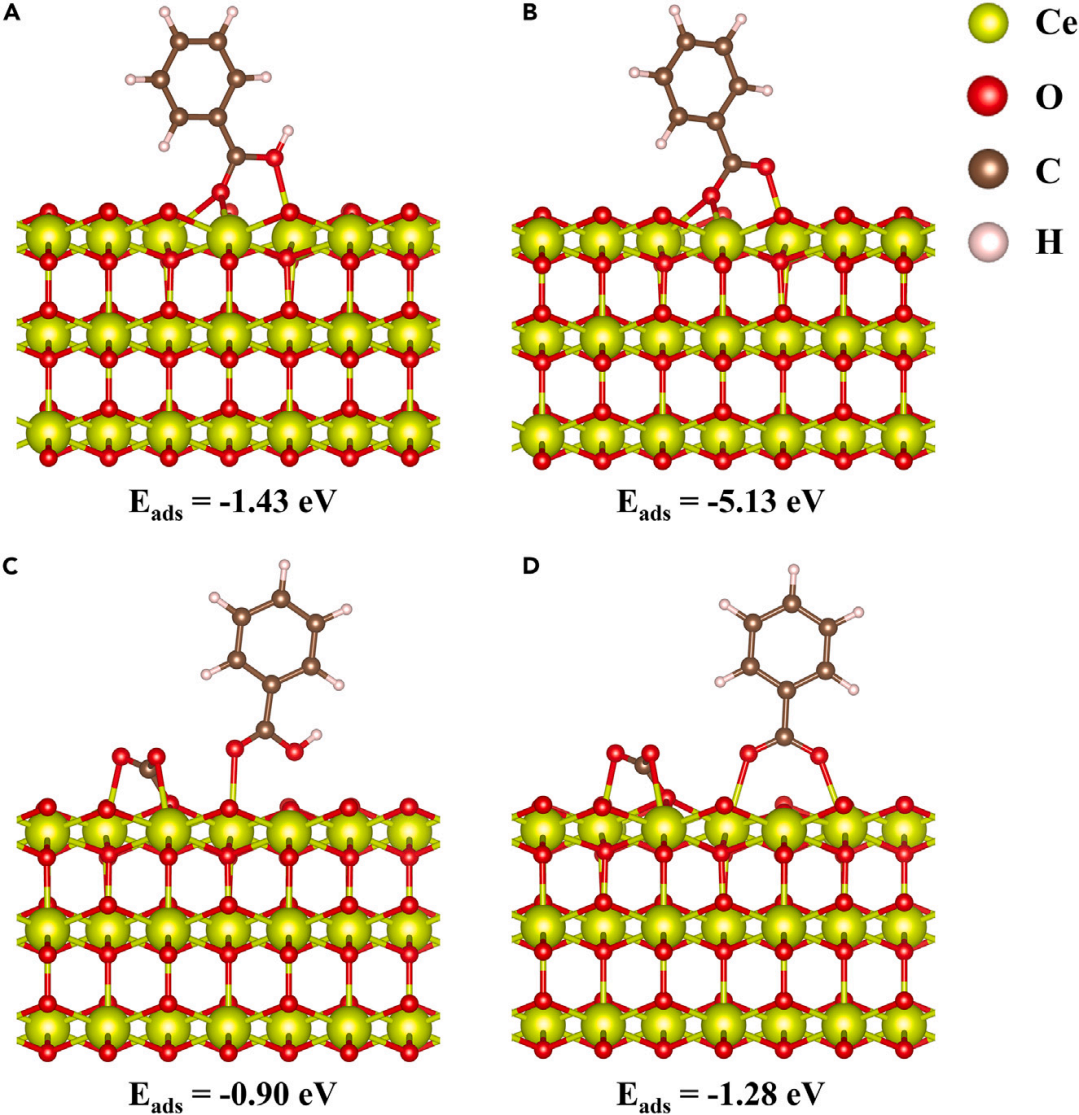
The optimized molecular adsorption states of BA on the CeO_2_ (111) surface (A and B) Before CO_2_ treatment. (C and D) After CO_2_ treatment.

To further understand the molecular adsorption states of BA on CeO_2_ before and after CO_2_ treatment, density functional theory (DFT) calculations were further performed. Two forms of adsorbed BA, molecular (PhCOOH∗) and benzoate (PhCOO∗), were examined on the (111) surface of CeO_2_ with O vacancy. The optimized adsorption states and adsorption energy (E_ads_) values are shown in Figure 4, respectively. The distinct difference of E_ads_ (−1.43 eV vs. −5.13 eV) demonstrates the much stronger adsorption of benzoate on raw CeO_2_ (111) surface (Figures 4A and 4B). Then CO_2_ was introduced to the CeO_2_ (111) to simulate the CO_2_ treatment catalysts. The decreased E_ads_ values of two adsorbates indicate the relatively moderate adsorption on CO_2_ adsorbed CeO_2_, and both the two forms are reasonable to exist because of the similar E_ads_ values (Figures 4C and 4D). These results are consistent with those from the DRIFTS and Raman tests.

### Effect of substrate adsorption state on reaction pathway

**Scheme 2:**
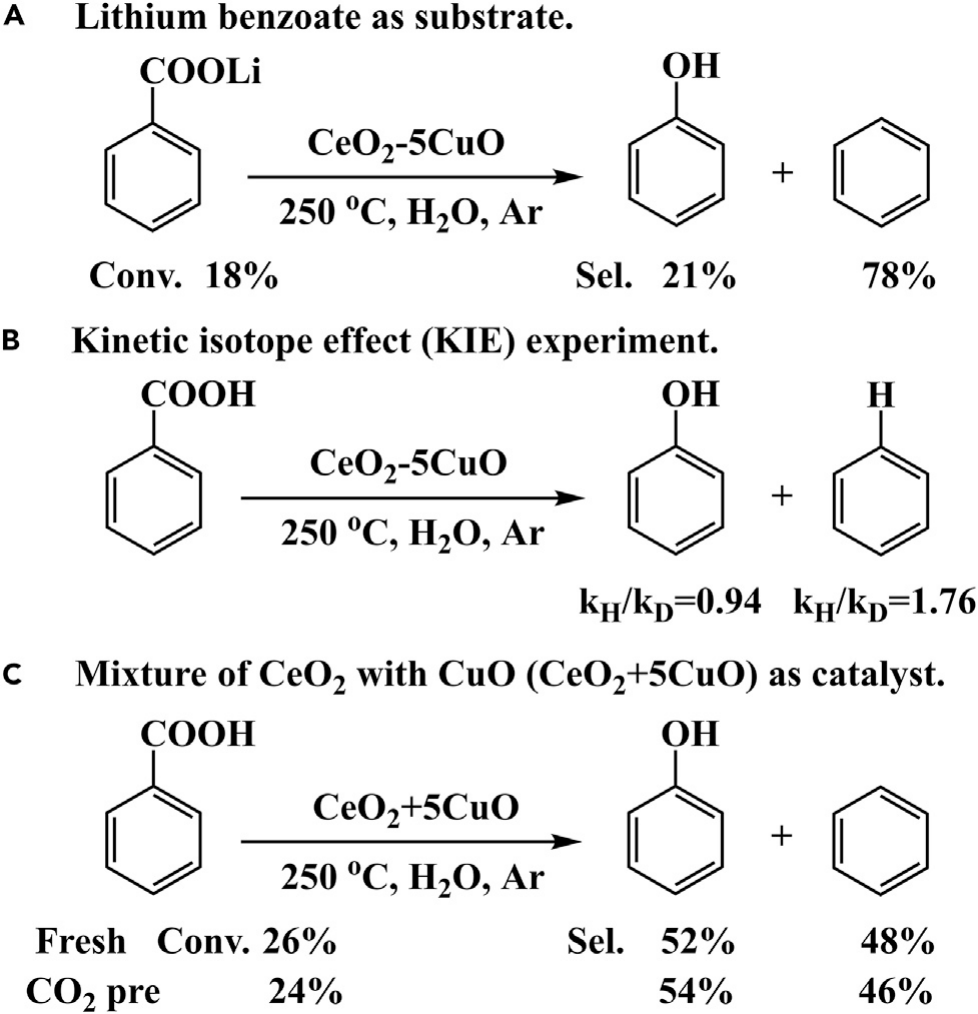
Control experiments (A) Lithium benzoate as substrate. (B) Kinetic isotope effect (KIE) experiment. (C) Mixture of CeO_2_ with CuO as catalyst.

Control experiments were carried out to clarify the influence of the adsorption state of BA on the subsequent reaction pathways (Scheme 2). The above catalytic performance and spectroscopy results suggest that the dissociation of carboxyl to form benzoate leads to the production of benzene. Therefore, lithium benzoate was used as the substrate, and the obviously increased selectivity of benzene (78%) confirmed our deduction (Scheme 2A). Kinetic isotope effect (KIE) experiments were also carried out (Scheme 2B). BA with deuterated carboxyl was reacted under standard conditions, in contrast to the undeuterated BA substrate. The k_H_/k_D_ = 1.76 of benzene indicated proton dissociation in carboxyl affecting the rate of decarboxylation, while the k_H_/k_D_ = 0.94 of phenol showed no obvious KIE in decarboxylative oxidation. Furthermore, a physical mixture of CeO_2_ with CuO (CeO_2_+5CuO) as the catalyst was tested (Scheme 2C). CO_2_ treatment of the physically mixed catalyst showed no influence on product selectivity. This phenomenon demonstrates the synergistic effect of CeO_2_ and CuO, adsorption and catalytic conversion, respectively.

**Scheme 3:**
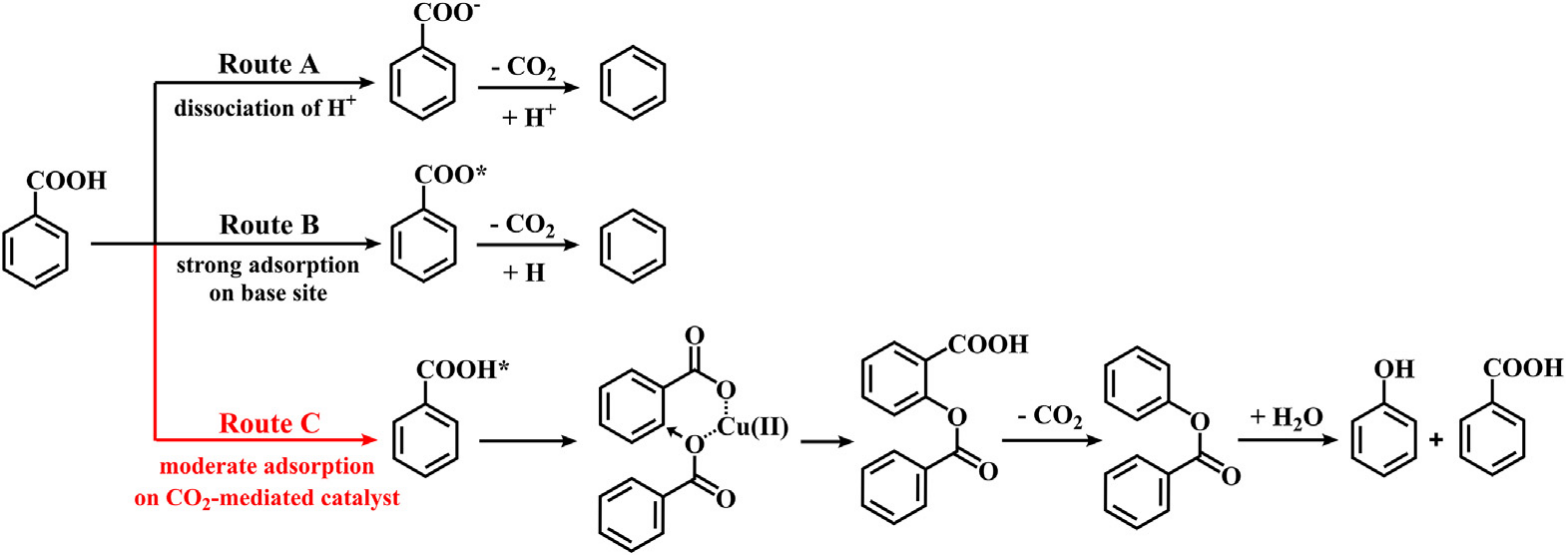
The proposed reaction pathway for decarboxylative oxidation of benzoic acid.

Based on the above results, the reaction pathway for this adsorption-regulated decarboxylative oxidation process was proposed (Scheme 3). Basically, the dissociation of carboxyl in BA to form benzoate promotes the decarboxylation to generate benzene, which was reported in previous work^31^^,^^32^^,^^33^^,^^34^ and also confirmed by our experiment results. The proton dissociation of BA in water at 250°C results in about 50% selectivity to benzene on CuO (Route A). The introduction of the CeO_2_ component achieves strong adsorption of BA on the catalyst, but the base sites on CeO_2_ lead to benzoate adsorption (Route B). The CO_2_-mediated catalysts exhibit moderate adsorption of BA, which is prone to generate the paddlewheel intermediate in the decarboxylative oxidation process (Route C).

### Synthesis of *meta*-substituted phenols from benzoic acids

**Scheme 4:**
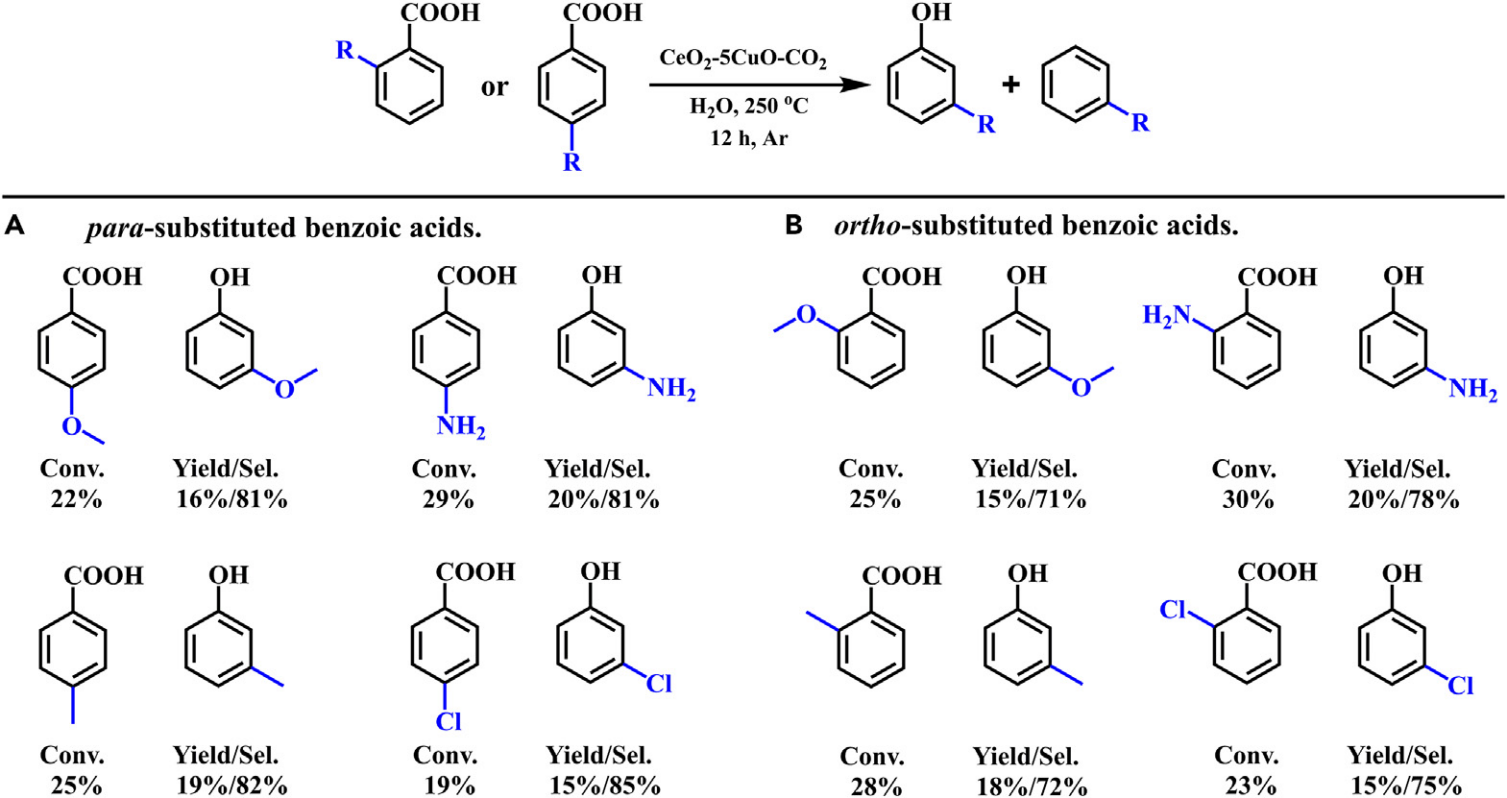
Synthesis of *meta*-substituted phenols from benzoic acids (A) *para*-substituted benzoic acids. (B) *ortho*-substituted benzoic acids. Reaction conditions: 1.75 mmol of Cu, 1 mmol benzoic acids, 20 mL H_2_O, 250°C, 12 h, Ar atmosphere.

According to Route C in Scheme 3, paddlewheel intermediate (*ortho* salicylate benzoate) was formed, which inspired us to synthesize the *meta*-substituted phenols from *para* or *ortho* substituted BAs. It is challenging to get *meta*-substituted phenols from conventional *meta*-functionalization of phenols because the *meta* C–H of phenols is not electronically activated.^55^ Therefore, decarboxylative oxidation of BAs becomes an alternative method. Based on our above results, the CO_2_-mediated CeO_2_-5CuO catalyst was applied to the synthesis of *meta*-substituted phenols from BAs. As illustrated in Scheme 4, BAs bearing electron-neutral, electron-donating, and electron-withdrawing substituents were selectively transformed into the *meta*-substituted phenols under standard conditions for 12 h. The *meta*-phenols could be produced from *para*-substituted BAs with more than 80% selectivity, or from *ortho*-substituted BAs with more than 70% selectivity. Most notably, substituted BAs, such as methoxy BAs, are also reported to be produced from lignin oxidative depolymerization, which provides a sustainable method to synthesize *meta*-substituted phenols.

**Figure 5:**
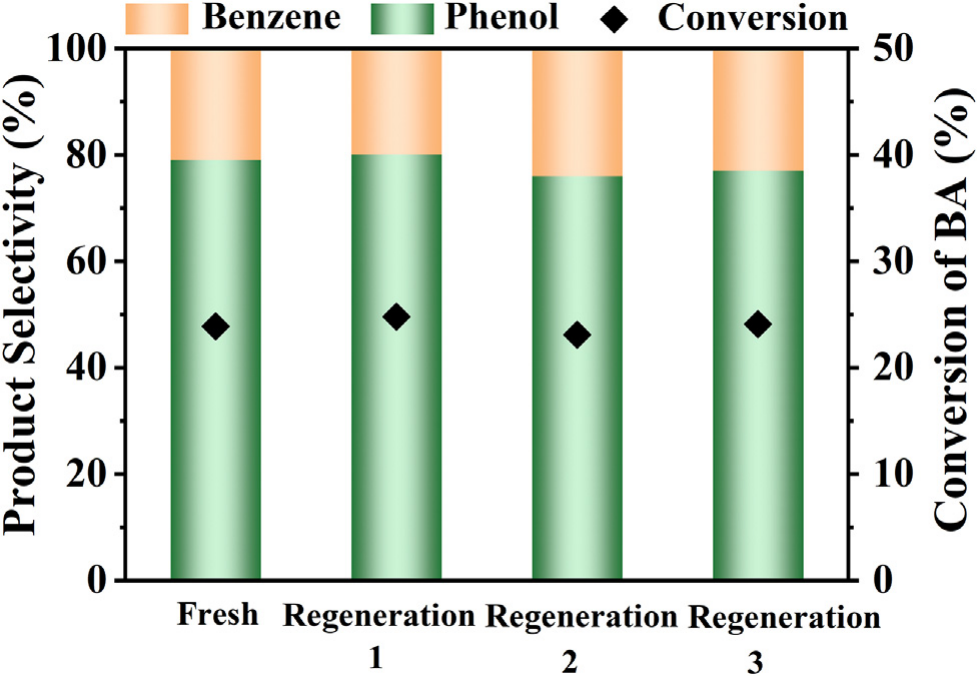
Reuse test of CeO_2_-5CuO-CO_2_ catalyst in decarboxylative oxygenation reaction of BA Reaction conditions: 1 mmol of Cu, 1 mmol BA, 20 mL H_2_O, 250°C, 12 h, Ar atmosphere. The used catalyst was regenerated by calcined under an air atmosphere at 400°C for 2 h and then treated under a CO_2_ atmosphere at 300°C for 1 h.

To be mentioned, the relatively low conversion was due to the deactivation of the catalyst and further XRD (Figure S5), XPS (Figure S6) and AES (Figure S7) studies of the used catalyst showed that it was caused by the reduction of Cu(II) to Cu(I) on the surface. Satisfyingly, the catalyst can be reactivated by simply calcined at 400°C in air. The reuse test showed that the CeO_2_-5CuO-CO_2_ catalyst could be used at least four times without an obvious decrease in activity and selectivity (Figure 5). The facile catalyst regeneration and stable catalytic performance make this strategy promising for industrial-scale decarboxylation of benzoic acid to produce *meta*-substituted phenols.

### Conclusion

In conclusion, we report a heterogeneous CO_2_-mediated CeO_2_-5CuO catalyst for decarboxylative oxidation of BAs, which facilitates the one-pot synthesis of *meta*-substituted phenols with more than 80% selectivity. This technology is based on a traceless directing group relay method. This selective procedure avoids the otherwise typical formation of benzene by-product via decarboxylation. DFT calculations and experimental investigations showed that the adsorption states of BA could be regulated by the introduction of CeO_2_ component and CO_2_ treatment, which are determinants for subsequent reaction pathways. Furthermore, CO_2_-mediated CeO_2_-5CuO catalyst after facile regeneration still has stable catalytic performance. Due to the abundance of BAs and the importance of phenols, the technology is expected to have broad applications in organic synthesis.

### Limitations of the study

The main content of our work is the synthesis of *meta*-substituted phenols from biomass-based BAs. However, the CeO_2_-5CuO catalyst needs to be regenerated under air atmosphere at 300°C after one time use under standard reaction conditions (250°C, 12 h, 1 mmol substrate). Further studies are ongoing to improve the stability of catalysts. In addition, the regeneration process may be facile to conduct in a fixed-bed reactor.

## STAR★Methods

### Key resources table

**Table:** 

REAGENT or RESOURCE	SOURCE	IDENTIFIER
**Chemicals, peptides, and recombinant proteins**		

Benzoic acid	Aladdin	CAS 65-85-0
Phenol	Aladdin	CAS 108-95-2
Benzene	Macklin	CAS 71-43-2
Oxalic acid	Macklin	CAS 144-62-7
Copper(II) nitrate	Aladdin	CAS 3251-23-8
Cerium(III) nitrate	Aladdin	CAS 10294-41-4
Magnesium(II) nitrate	Aladdin	CAS 13446-18-9
Zirconium(IV) nitrate	Aladdin	CAS 13746-89-9
Aluminum(III) nitrate	Aladdin	CAS 7784-27-2
Manganese(II) nitrate	Aladdin	CAS 10377-66-9

**Software and algorithms**		

Origin Pro 2019	Origin Lab Corporation	Massachusetts, USA
Density functional theory	Materials Studio	BIOVIA, USA

**Other**		

Fourier transform infrared spectroscopy	Thermo Fisher Scientific	IS50
Raman spectroscopy	Bruker Optics	Senterra
Gas chromatograph	Agilent	7890B
Gas chromatograph-mass spectrometry	Agilent	7890A-5975C
X-ray diffraction	Malvern Panalytical	Empyream
X-ray photoelectron spectroscopy	Thermo Fisher Scientific	Escalab 250 Xi+
Transmission electronic microscopy	JEOL	JEM-2100

### Resource availability

#### Lead contact

Further information and requests for resources and reagents should be directed to and will be fulfilled by the lead contact, Professor Xiaojun Shen (shenxiaojun@bjfu.edu.cn).

#### Materials availability

This study did not generate new unique reagent.

### Methods details

#### Chemicals and catalysts

All organic chemicals and metal nitrate precursors were purchased and used without further purifications. MO-CuO catalysts were synthesized by the co-precipitation method. Oxalic acid was used as a precipitating agent. The *x*MO-*y*CuO means the molar ratio of M/Cu in the mixed oxides is *x*:*y*, where M represents metal elements, including Mg, Ce, Zr, Al, Mn. The catalyst with CO_2_ pretreatment was labeled as *x*MO-*y*CuO-CO_2_.

Taking the synthesis procedure of CeO_2_-5CuO catalyst as an example: 0.02 mol Cu(NO_3_)_2_ and 0.004 mol Ce(NO_3_)_3_ precursors were dissolved in 100 mL ethanol. The mixed solution of precursors was then added into 0.5M oxalic acid in ethanol solution under vigorous stirring. After 30 min reaction, the resultant solid was separated by centrifugation, followed by washing with ethanol and drying overnight at 80°C. The obtained solid was calcined under an O_2_/N_2_ atmosphere at 400°C for 2 h. The MO-CuO catalysts with other metal oxides and ratios were also prepared by a similar procedure. The CO_2_ pretreatment of MO-CuO catalysts (MO-CuO-CO_2_) was calcined under a CO_2_ atmosphere at 300°C for 1 h.

#### Catalyst characterization

Transmission electron micrographs (TEM) were obtained on a JEM-2100 microscope operated at 200 kV. The samples were suspended in ethanol, and a few drops of the suspension were dried to the TEM grid for TEM measurement. X-ray diffractograms of the samples were obtained on a Malvern Panalytical Empyream Powder X-ray diffractometer with Cu Kα radiation. The measurement was operated at 40 kV and scanning 2θ from 5° to 80° with a step of 0.013°. The signal was collected by a pixel 1D detector, and the data were analyzed by comparison with reference patterns in the database (PDF2-2004). X-ray photoelectron spectra (XPS) were recorded on a Thermo Scientific Escalab 250 Xi equipped with a monochromatic Al Kα X-ray radiation source (hν = 1486.6 eV). The C 1s peak was used as the reference at 284.8 eV. Diffuse reflectance infrared Fourier transform spectroscopy (DRIFTS) spectra of adsorbed BA on catalysts were collected in an *in-situ* reaction cell on a Thermo Scientific Nicolet iS50 equipped with an MCT detector. Prior to analysis, the catalyst was added to the benzoic acid aqueous solution at 100°C for 1 h and then washed with ethanol three times. The obtained solid was dried at 80°C overnight under a vacuum to get BA adsorbed catalyst. DRIFT spectra were recorded after the reaction cell was purged with Ar for 10 min to remove the air. The background and catalyst sample spectra were scanned 64 times at one set with a 4 cm^−1^ resolution. Raman spectra were collected with a 532 nm constant-wave laser (Bruker Optics) that served as the excitation source.

#### Catalytic evaluation

Catalytic reactions were carried out in a 50 mL stainless steel batch reactor from Beijing Century Senlong Experimental Apparatus Co. Ltd. The reactor was equipped with a magnetic stirrer, a thermocouple, a pressure gauge, and a programmable controller. In a typical run, 1 mmol of benzoic acid, 20 mL of H_2_O, and 1 mmol of Cu-based catalyst were loaded into the reactor. The reactor was purged with Ar for 10 min to remove the air. The reactor was then heated to 250°C and kept for a specified reaction time while the content was stirred at a rate of 600 rpm. After the reaction, the liquid was extracted with 20 mL ethyl acetate three times and 73.8 mg of n-dodecane as an internal standard was added into the organic phase. The reaction products were identified by GC-MS (Agilent 7890A-5975C) and quantified by GC (Agilent 7890B) with a flame ionization detector (FID) using an HP-INNOWax column.

The conversion of benzoic acid (conv.), selectivity of product (sel.) and product yield were calculated by Equations 1, 2 and 3, respectively. In these equations, *n*_initial BA_ and *n*_final BA_ are the molar amount of benzoic acid before and after reaction. *n*_product i_ is the molar amount of product i in the reaction mixture. *n*_*total*_ _product_ is the total molar amount of products in the reaction mixture.

(Equation 1) $$conversion\;\left(\%\right)=\frac{n_{initial\;BA}-n_{final\;BA}}{n_{initial\;BA}}\times 100\%$$

(Equation 2) $$selectivity\;\left(\%\right)=\frac{n_{product\;i}}{n_{total\;product}}\times 100\%$$

(Equation 3) $$yield\;\left(\%\right)=\frac{n_{product\;i}}{n_{initial\;BA}}\times 100\%$$

#### Computational method

We have employed the Vienna Ab Initio Package (VASP)^56^^,^^57^ to perform all the density functional theory (DFT) calculations within the generalized gradient approximation (GGA) using the PBE^58^ formulation. We have chosen the projected augmented wave (PAW) potentials^59^^,^^60^ to describe the ionic cores and take valence electrons into account using a plane wave basis set with a kinetic energy cutoff of 450 eV. Partial occupancies of the Kohn−Sham orbitals were allowed using the Gaussian smearing method and a width of 0.05 eV. The on-site corrections (DFT+U) have been applied to the 4f electron of Ce atoms (U_eff_ = 4.5 eV) by the approach from Dudarev et al.^61^ The electronic energy was considered self-consistent when the energy change was smaller than 10^−5^ eV. A geometry optimization was considered convergent when the force change was smaller than 0.02 eV/Å. Grimme’s DFT-D3 methodology^62^ was used to describe the dispersion interactions.

The equilibrium lattice constant of cubic CeO_2_ unit cell was optimized, when using an 11 × 11×11 Monkhorst-Pack k-point grid for Brillouin zone sampling, to be a = 5.479 Å. We then use it to construct a CeO_2_ (111) surface model with *p* (4 × 4) periodicity in the X and Y directions and 3 stoichiometric layers in the Z direction by vacuum depth of 15 Å in order to separate the surface slab from its periodic duplicates. This model comprises of 48 Ce and 96 O atoms. Model 1 was built by removing one O atom on the outmost layer in order to create an O vacancy. Model 2 was built by adding one CO_3_ in the O vacancy of model 1. During structural optimizations, the Γ point in the Brillouin zone was used for k-point sampling, and the bottom two stoichiometric layers were fixed while the rest were allowed to fully relax. The adsorption energy (E_ads_) of adsorbate A was defined as

$$E_{ads}=E_{A/surf}-E_{surf}-E_{A\left(g\right)}$$

where E_A/surf_, E_surf_ and E_A(g)_ are the energy of adsorbate A adsorbed on the polyimide, the energy of clean polyimide, and the energy of isolated A molecule in a cubic periodic box with a side length of 20 Å and a 1 × 1×1 Monkhorst-Pack k-point grid for Brillouin zone sampling, respectively.

## Data Availability

• The published article includes all datasets generated or analyzed during this study. • Any additional information required to reanalyze the data reported in this paper is available from the lead contact upon request. • This paper does not report original code.
