# Supplementary material for: Selective synthesis of meta-phenols from bio-benzoic acids via regulating the adsorption state
Source: iScience. 2023 Jul 25;26(8):107460. doi: 10.1016/j.isci.2023.107460 (PMC10428116; doi:10.1016/j.isci.2023.107460)
Supplement: Document S1. Figure S1–S8 [file mmc1.pdf]

iScience, Volume *26*

Supplemental information

Selective synthesis of *meta*-phenols from bio-benzoic acids via regulating the adsorption state

Xinze Du, Yumei Liu, Huixiang Li, Shenglin Liu, and Xiaojun Shen

Table of contents

1. Catalytic reactions

1.1 Product yield of decarboxylative oxidation reactions of benzoic acid **(Figure S1)** S2

1.2 Time profile of decarboxylative oxidation reactions of benzoic acid **(Figure S2)** S3

1.3 Identification of gas product **(Figure S3)** S4

2. Characterization of catalysts

2.1 XPS spectra of CeO_2-5CuO catalyst **(Figure S4)** S5

2.2 XRD spectra of used catalyst **(Figure S5)** S6

2.3 XPS spectra of the used catalyst **(Figure S6)** S7

2.4 Auger electron spectroscopy spectra of the used catalyst **(Figure S7)** S8

2.5 XPS spectra of CeO_2-5CuO-CO_2 catalyst **(Figure S8)** S9

1. Catalytic decarboxylative oxidation reactions of benzoic acid

1.1 Product yield of decarboxylative oxidation of benzoic acid

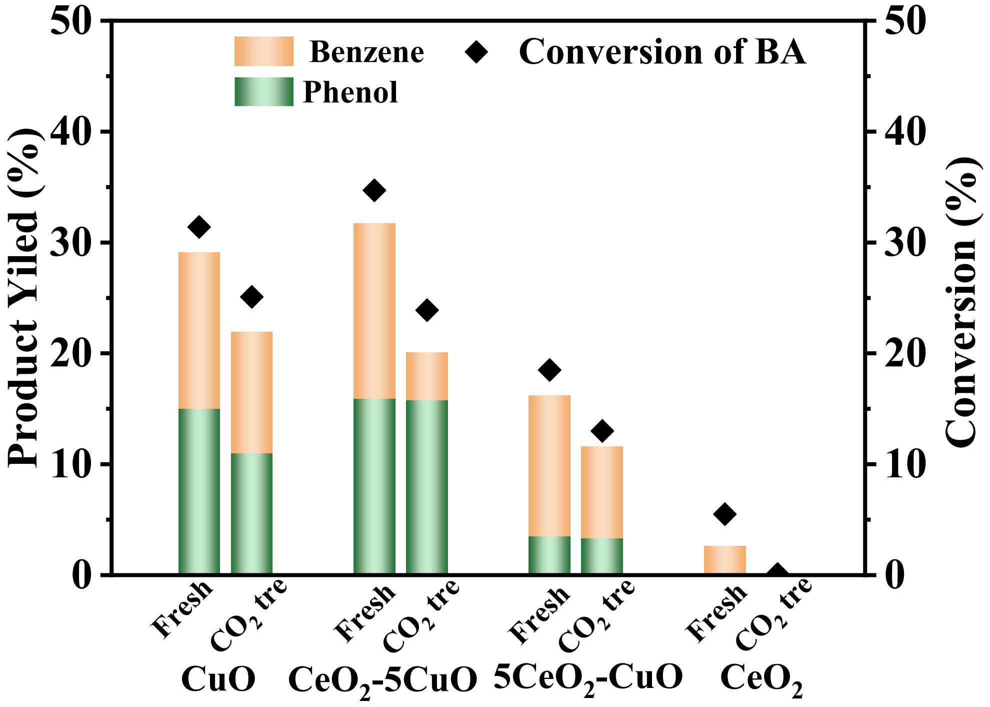

Figure S1. Catalytic performances of CeO_2-CuO catalysts in decarboxylative oxygenation reaction of BA. Related to Figure 1. Reaction conditions: 1 mmol of Cu, 1 mmol BA, 20 mL H_2O, 250 °C, 12 h, Ar atmosphere.

1.2 Time profile of decarboxylative oxidation reactions of benzoic acid

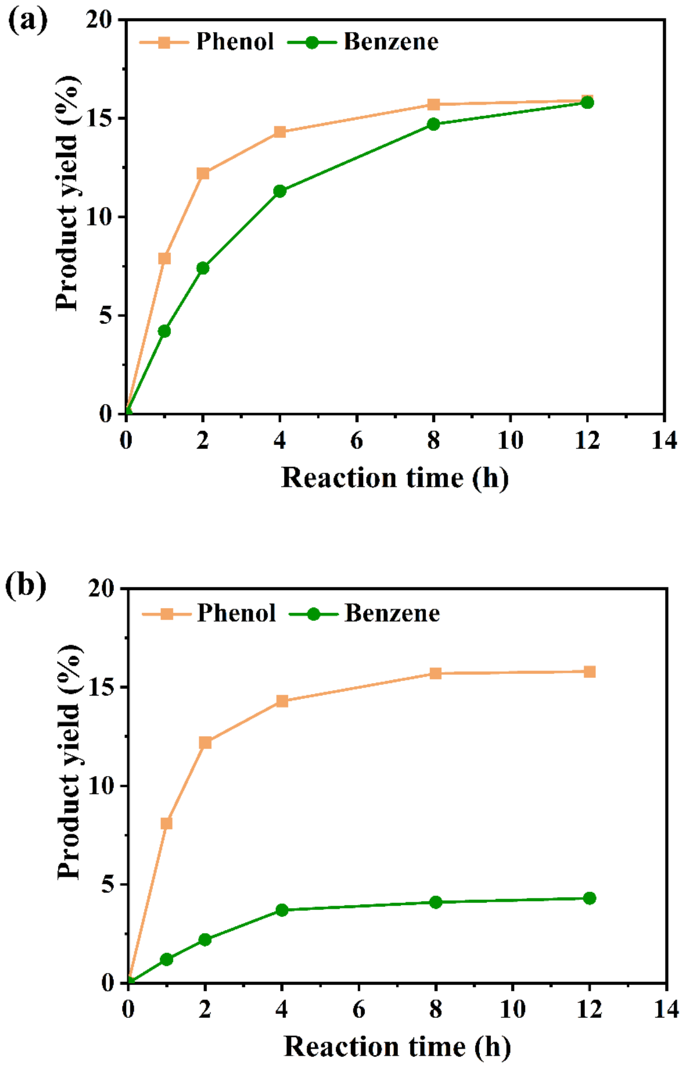

Figure S2. Time profile of decarboxylative oxidation reactions of benzoic acid catalyzed by: (a) CeO_2-5CuO; (b) CeO_2-5CuO-CO_2. Related to Figure 1. Reaction conditions: 1 mmol of Cu,1 mmol BA, 20 mL H_2O, 250 °C, Ar atmosphere.

1.3 Identification of gas product

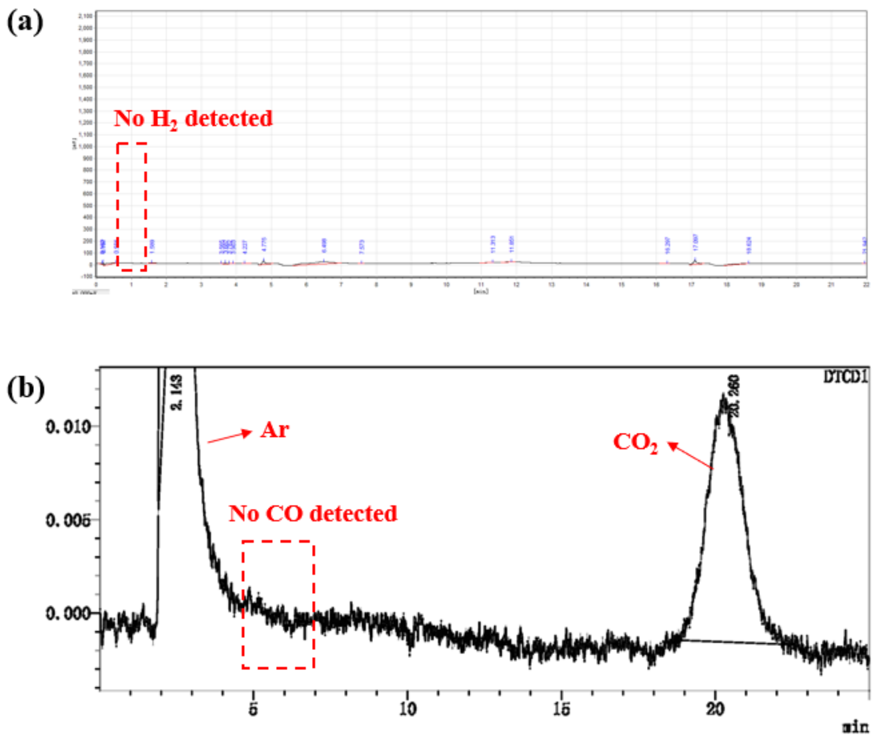

Figure S3. Gas product in decarboxylative oxygenation reaction of BA. Related to Figure 1. Reaction conditions: 1 mmol of Cu in CeO_2-5CuO-CO_2, 1 mmol BA, 20 mL H_2O, 250 °C, 12 h, Ar atmosphere.

2. Characterization of catalysts

2.1 XPS spectra of CeO_2-5CuO catalyst

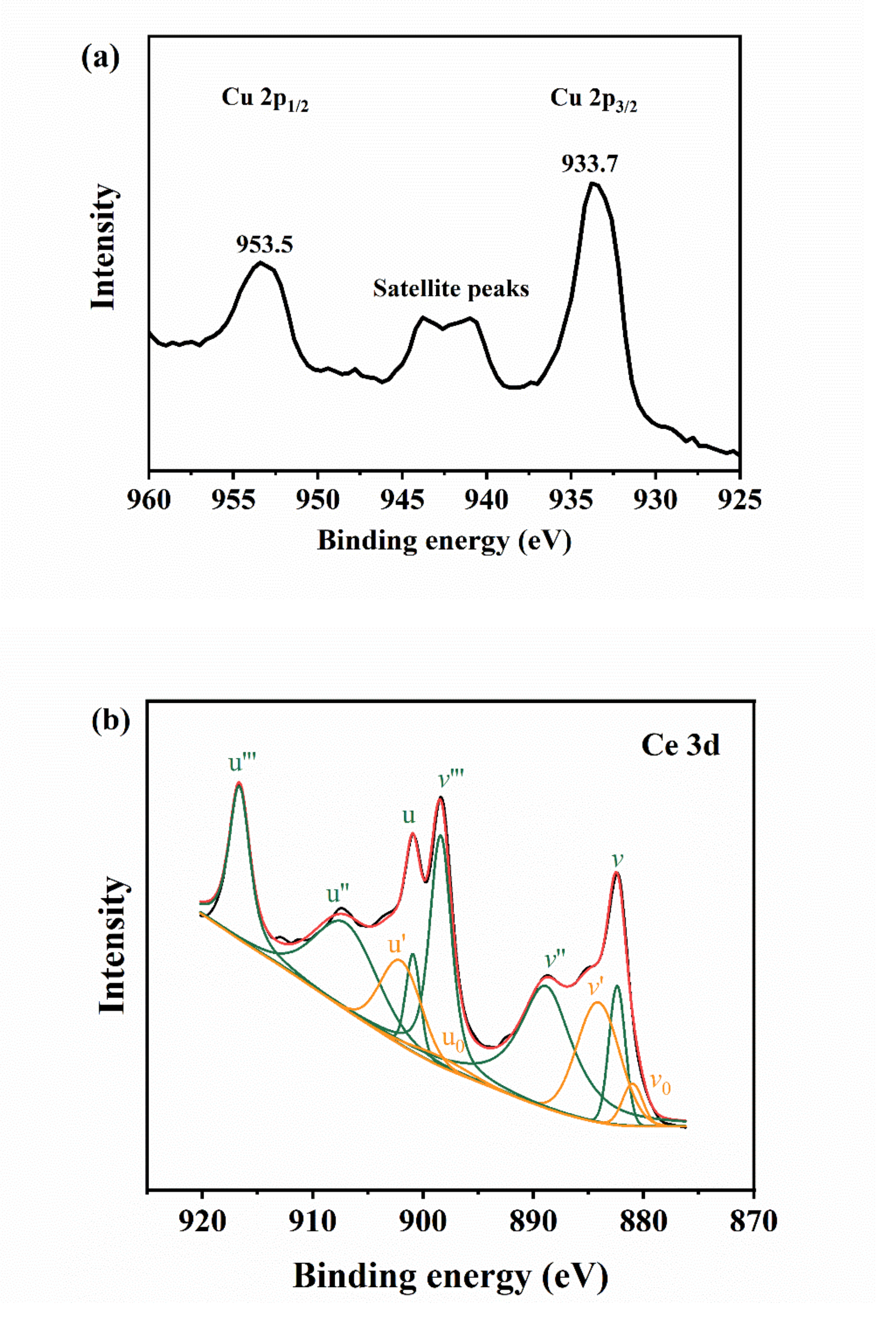

Figure S4. XPS spectra of CeO_2-5CuO catalyst: (a) Cu 2p; (b) Ce 3d. Related to Figure 2.

2.2 XRD spectra of used CeO_2-5CuO-CO_2 catalyst

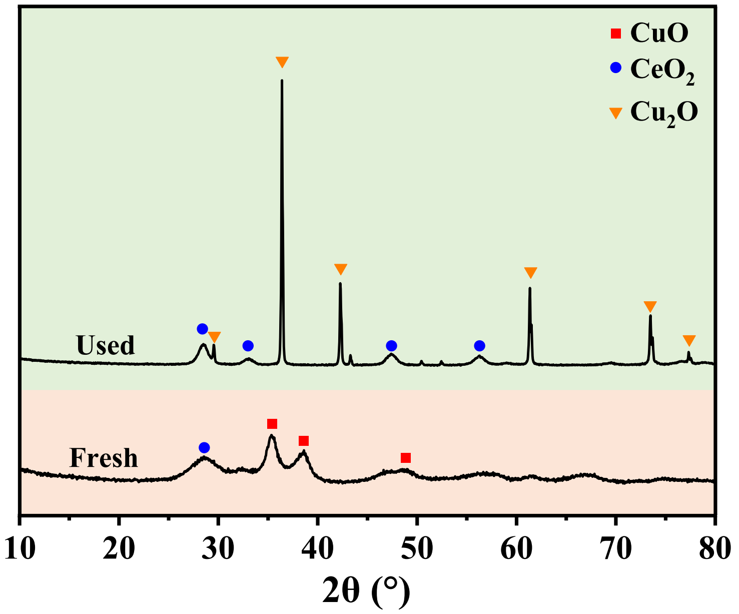

Figure S5. XRD spectra of used CeO_2-5CuO-CO_2 catalyst. Related to Figure 2.

2.3 XPS spectra of the used catalyst

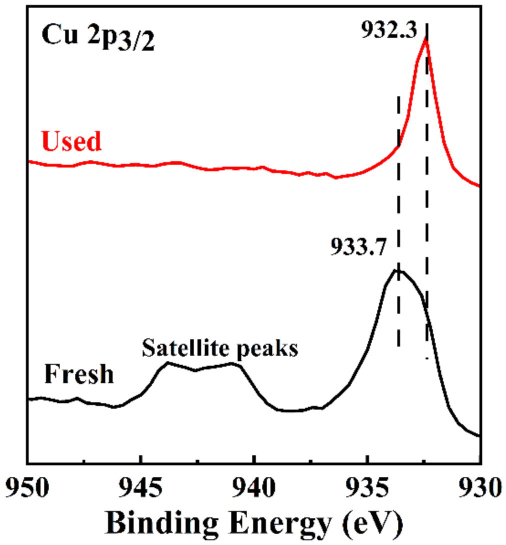

Figure S6. XPS spectra of used CeO_2-5CuO-CO_2 catalyst. Related to Figure 2.

2.4 Auger electron spectroscopy spectra of the used catalyst

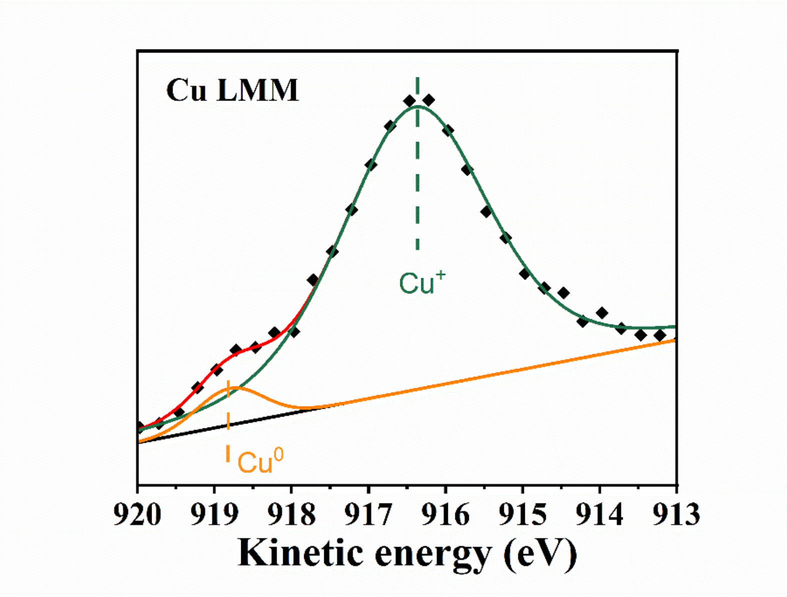

Figure S7. Auger electron spectroscopy (AES) spectra of used CeO_2-5CuO-CO_2 catalyst. Related to Figure 2.

2.5 XPS spectra of CeO_2-5CuO-CO_2 catalyst

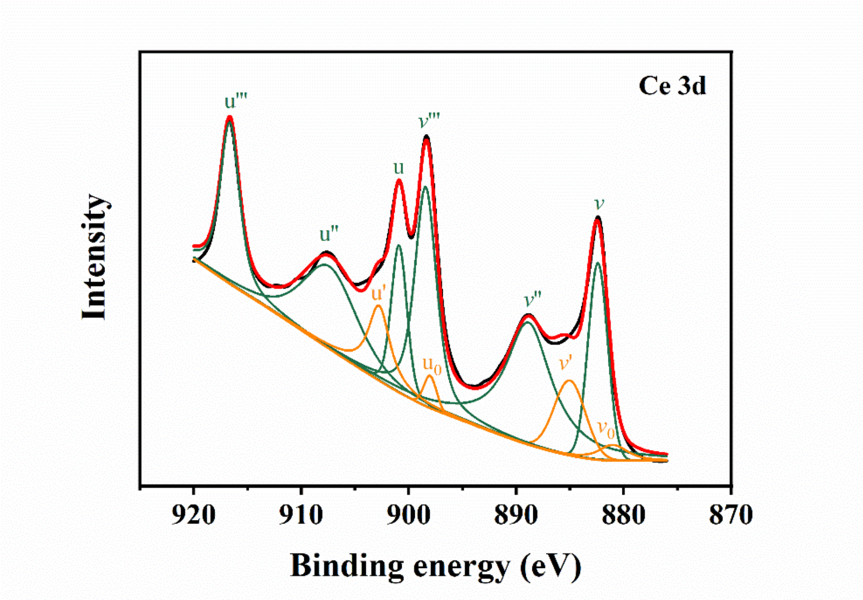

Figure S8. Ce 3d XPS spectra of CeO_2-5CuO-CO_2 catalyst. Related to Figure 2.
